# EMT and inflammation: inseparable actors of cancer progression

**DOI:** 10.1002/1878-0261.12095

**Published:** 2017-06-26

**Authors:** Meggy Suarez‐Carmona, Julien Lesage, Didier Cataldo, Christine Gilles

**Affiliations:** ^1^ National Center for Tumor Diseases (NCT) – University Hospital Heidelberg Germany; ^2^ Laboratory of Tumor and Development Biology GIGA‐Cancer University of Liège Belgium; ^3^ Inserm UMR‐S 903 SFR CAP‐Santé University of Reims Champagne‐Ardenne (URCA) France

**Keywords:** cancer, chemokines, cytokines, epithelial‐to‐mesenchymal transition, inflammation, metastasis

## Abstract

Tumors can be depicted as wounds that never heal, and are infiltrated by a large array of inflammatory and immune cells. Tumor‐associated chronic inflammation is a hallmark of cancer that fosters progression to a metastatic stage, as has been extensively reviewed lately. Indeed, inflammatory cells persisting in the tumor establish a cross‐talk with tumor cells that may result in a phenotype switch into tumor‐supporting cells. This has been particularly well described for macrophages and is referred to as tumor‐associated ‘M2’ polarization. Epithelial‐to‐mesenchymal transition (EMT), the embryonic program that loosens cell–cell adherence complexes and endows cells with enhanced migratory and invasive properties, can be co‐opted by cancer cells during metastatic progression. Cancer cells that have undergone EMT are more aggressive, displaying increased invasiveness, stem‐like features, and resistance to apoptosis. EMT programs can also stimulate the production of proinflammatory factors by cancer cells. Conversely, inflammation is a potent inducer of EMT in tumors. Therefore, the two phenomena may sustain each other, in an alliance for metastasis. This is the focus of this review, where the interconnections between EMT programs and cellular and molecular actors of inflammation are described. We also recapitulate data linking the EMT/inflammation axis to metastasis.

**Abbreviations**
CAFs: cancer‐associated fibroblasts
CTCs: circulating tumor cells
DCregs: regulatory dendritic cells
EMT: epithelial‐to‐mesenchymal transition
EMT‐TF: EMT transcription factor
MMPs: matrix metalloproteinases
TAMs: tumor‐associated macrophages
Tregs: regulatory T cells

## 1 Introduction

While acute, transitory inflammation is an essential actor of tissue damage control and repair, tumor‐associated inflammation – which occurs in virtually all tumors – is of a chronic, unresolved type (Pesic and Greten, 2016) that fosters tumor progression. During tumorigenesis, cancer cells, innate immune cells [such as dendritic cells or tumor‐associated macrophages (TAMs)] and activated resident cells [such as cancer‐associated fibroblasts (CAFs) or endothelial cells] produce a variety of cytokines and chemokines in response to the danger signals originating from the tumor. These soluble factors drive the recruitment of massive amounts of additional bone marrow‐derived innate immune cells, which fuel the so‐called cytokine storm (Crusz and Balkwill, 2015). This prolonged reaction favors tumor cell survival and proliferation, immunosuppression (by the inhibition of effector immune cells and the accumulation of myeloid suppressive cells) and angiogenesis (Becht *et al*., 2016). Promisingly, multiple anti‐inflammatory agents are under development and/or under clinical testing currently in chemoprevention trials (Crusz and Balkwill, 2015).

The understanding that tumor cells are genetically and phenotypically very heterogeneous has further stimulated studies aiming at deciphering how different tumor cell phenotypes may relate to tumor‐associated inflammation. During the metastatic progression of epithelial tumors, tumor cells indeed undergo phenotypic changes, essentially driven by environmental stimuli, allowing the tumor cells to adapt to the different microenvironment encountered (adjacent stroma, blood, or colonized organs). Epithelial‐to‐mesenchymal transition (EMT) appears today as a major actor modulating these phenotypic conversions. Although two recent studies (Fischer *et al*., 2015; Zheng *et al*., 2015) have revived the debate about the universal requirement of EMT in the metastasis process, the current dogma is that EMT processes might be involved in the initial steps of the metastatic cascade, including tumor invasion, intravasation, and micrometastases formation. This has been supported by multiple *in vitro* and *in vivo* functional data, as well as by correlative data in human samples (Diepenbruck and Christofori, 2016). The acquisition of EMT‐like changes in tumor cells has been extensively studied and implies increased invasive properties, resistance to DNA damage‐ and chemotherapy‐induced apoptosis, immunosuppression, and the acquisition of stem‐like features. EMT transcriptomic signatures are found highly associated with groups of patients with poorer outcome in multiple cancer entities including breast cancer (Jang *et al*., 2015), colorectal cancer (Roepman *et al*., 2014), head and neck cancer (Pan *et al*., 2016), or malignant pleural mesothelioma (de Reynies *et al*., 2014). EMT‐associated signaling pathways have lately been considered as therapeutic targets, as recently shown in a murine pancreatic cancer model (Subramani *et al*., 2016). In this study, metastasis was successfully hampered by the use of nimbolide, a drug that, among other effects, reduced EMT via the induction of excessive production of reactive oxygen species (ROS). EMT signaling pathways have also been targeted in breast cancer *in vitro* models (Yu *et al*., 2016), or even in clinical settings (Marcucci *et al*., 2016).

Increasing literature data have thus emphasized a link between cancer‐associated EMT and chronic inflammation. Indeed, inflammatory mediators (soluble factors, oxidative stress, or hypoxia, for example) can foster the acquisition of EMT‐like features in cancer cells (Lopez‐Novoa and Nieto, 2009) (Table 1). Conversely, these cancer cells can produce a higher amount of proinflammatory mediators, such as cytokines, chemokines, and matrix metalloproteinases (MMPs), which fuel the cancer‐related, smoldering inflammation. In this review, we highlight most recent data including *in vitro* and *in vivo* models describing the connections between EMT signaling pathways and (i) innate immune cells and (ii) soluble mediators of inflammation (inflammatory cytokines and chemokines). Finally, we present some *in vivo* functional studies and human correlative data, associating EMT and inflammation with tumor progression and metastasis.

**Table 1:** Soluble actors of inflammation and their described effect on EMT activation in cancer cells *in vitro*

Soluble factors	Cellular origin	Evidence of EMT pathway activation in cancer cells	Cell line origin	References
TNF‐α	Pleiotropic (mostly macrophages)	Increased invasive properties E‐cadherin drop N‐cadherin and vimentin expression	Hepatocellular carcinoma	Zhu *et al*. (2016)
		Slug and ZEB1 expression Increased cell migration E‐cadherin drop	Renal cell carcinoma	Sun *et al*. (2016)
		Increased invasion and migration E‐cadherin drop Vimentin and N‐cadherin expression	Papillary thyroid cancer	Lv *et al*. (2015)
		Altered mRNA expression of Snail1, ZEB1, E‐cadherin, fibronectin, vimentin, TGM2	Breast cancer	Cohen *et al*. (2015), Elghonaimy *et al*. (2016)
		E‐cadherin drop Vimentin expression Increased migration	Colorectal cancer	Bates and Mercurio (2003), Bhat *et al*. (2016)
TGF‐β	Ubiquitous	Elongated cell shape Low E‐to‐N‐cadherin ratio, vimentin expression Smad‐dependent signaling Smad‐independent signaling	Breast cancer	Johansson *et al*. (2015), Moustakas and Heldin (2016), Pang *et al*. (2016)
		Increased migration and invasion E‐cadherin drop N‐cadherin expression Snail1, Snail2, and ZEB1 gene expression	Lung cancer	Wu *et al*. (2016b)
		Morphological changes E‐cadherin drop Vimentin expression Gene and protein expression of EPCAM (and stemness markers) Invasion and migration	Hepatocellular carcinoma	Malfettone *et al*. (2017)
IL‐1β	Macrophages, tumor cells	ZEB1 expression E‐cadherin drop Morphological changes Increased invasiveness	Colorectal cancer	Li *et al*. (2012b)
		Snail1 and Slug expression Vimentin E‐cadherin drop Increased migration	Oral cancer (cancer cell lines and dysplastic oral keratinocytes)	Lee *et al*. (2015)
IL‐6	T cells, macrophages, tumor cells	Morphological changes JAK2/STAT3/Snail1 pathway activation E‐cadherin drop Vimentin expression Increased migration	Head and neck cancer	Yadav *et al*. (2011)
		JAK2/STAT3/Twist1 activation E‐cadherin drop, Vimentin, N‐cadherin, and fibronectin expression	Breast cancer	Kim *et al*. (2016), Sullivan *et al*. (2009)
		Altered mRNA expression of vimentin, Snail1, Slug, and ZEB1 Increased invasive properties	Colorectal cancer	Rokavec *et al*. (2014)
		Multiple markers (E‐cadherin, N‐cadherin, Twist, vimentin, MMP9, VEGF, TGF‐β) Increased migratory properties	NSCLC	Lee *et al*. (2016)
IL‐8	T cells, macrophages, tumor cells	ZEB1, Snail1, and Slug activation Increased migratory properties	Thyroid carcinoma	Visciano *et al*. (2015)
		JAK2/STAT3/Snail1 activation E‐cadherin drop N‐cadherin expression Increased migratory properties	Hepatocellular carcinoma	Fu *et al*. (2015)
		E‐cadherin gene repression	Nasopharyngeal carcinoma	Zhang *et al*. (2016e)
		Morphological chances Increased migratory and invasive properties E‐cadherin drop N‐cadherin, vimentin, fibronectin expression	Breast cancer	Ji *et al*. (2016)
		E‐cadherin drop Vimentin and fibronectin expression Increased invasive properties	NSCLC	Fernando *et al*. (2016)
		E‐cadherin drop Increased migratory properties	Ovarian cancer	Yin *et al*. (2015)
CCL2	Monocytes, macrophages, dendritic cells	Only studied in combination with IL‐6 Twist expression Increased migration E‐cadherin drop Vimentin and fibronectin expression	NSCLC	Chen *et al*. (2015b)
		E‐cadherin drop, increased Snail1 expression STAT3 activation MMP9 expression Increased migration	Prostate cancer	Izumi *et al*. (2013)
		Increased invasion and migration E‐cadherin/vimentin switch, increased N‐cadherin Increased Snail1 and Twist gene expression Increased MMP9 expression	Bladder cancer	Rao *et al*. (2016)
CCL5	Cancer stem cells, exhausted T cells, adipocytes	E‐cadherin drop Expression of vimentin, Snail1, Slug Increased invasive properties	Ovarian cancer	Long *et al*. (2015)
		E‐cadherin drop Expression of vimentin, Snail1	Colorectal cancer	Halama *et al*. (2016)
		Migratory and invasive properties only	Triple‐negative breast cancer	D'Esposito *et al*. (2016)
CCL18	Macrophages, tumor cells	Migratory and invasive properties only	Ovarian cancer	Wang *et al*. (2016)
		E‐cadherin drop, Vimentin expression Migratory and invasive properties	Breast cancer	Su *et al*. (2014)
		Increased invasive properties Snail1 gene expression	Pancreatic cancer	Meng *et al*. (2015)
CCL20 (+IL‐8)	Lymphocytes	Morphological changes E‐cadherin drop Vimentin expression Increased migration	Colorectal cancer	Cheng *et al*. (2014), Kapur *et al*. (2016)
CCL21	Lymphocytes	ERK1/2/NF‐κB signaling Increased invasive properties E‐cadherin drop N‐cadherin and MMP9 expression	Pancreatic cancer	Zhang *et al*. (2016c)
IL‐23	Th17 lymphocytes	Wnt/β‐catenin pathway activation, GSK3β stabilization Snail1 and Slug gene expression E‐cadherin/vimentin switch	Esophageal cancer	Chen *et al*. (2015)
IL‐17	Th17 lymphocytes	Snail1 and Slug expression E‐cadherin/vimentin switch	Prostate cancer	Zhang *et al*. (2016d)
		E‐cadherin/vimentin switch Increased invasive properties	Gastric cancer	Jiang *et al*. (2017)

## 2 EMT and the cellular actors of inflammation

Inflammatory cells in a tumor can be of multiple origins. These cells comprise activated resident cells, such as endothelial cells or CAFs or resident macrophages (TAMs) or dendritic cells. In addition, the tumor milieu recruits bone marrow‐derived cells, mostly neutrophils, macrophages, and immature, immunosuppressive myeloid cells called myeloid‐derived suppressive cells (MDSCs). In this section, we review the interconnection between EMT and recruitment and/or polarization of such cells.

### 2.1 EMT and myeloid cells

#### 2.1.1 TAMs

Among immune cells infiltrating solid tumors, TAMs are most likely the most abundant cell type as they make up to 50% of the tumor mass (Solinas *et al*., 2009). Even though macrophages should be able to kill tumor cells, provided they get the appropriate activation signals, the chronically inflamed/immunosuppressive microenvironment most often polarizes TAMs into tumor‐supporting cells (schematically categorized in ‘M2‐like macrophages’) that promote extracellular matrix remodeling, angiogenesis, immunosuppression, and foster the acquisition of invasive properties by cancer cells by secreting various soluble factors (Becht *et al*., 2016).

The global numbers of TAMs have been correlated with EMT‐like features in several cancer entities. This has been reported in a cohort of 178 patients with gastric cancer, where CD68‐positive cell density is associated with the expression of EMT features in cancer cells (E‐cadherin loss and vimentin *de novo* expression). This was also demonstrated in an independent study using another marker of TAMs, CD163. In that study, high intratumoral CD163 expression was found to be correlated with E‐cadherin loss (Yan *et al*., 2016). Global density of macrophages also relates to EMT features in non‐small‐cell lung cancer carcinoma (NSCLC) (Bonde *et al*., 2012) or head and neck cancer (Hu *et al*., 2016).

Spatial association of cancer cells with EMT features and macrophages has also been described in a murine model of terato‐carcinoma. Tumor areas rich in macrophages indeed contain more tumor cells displaying E‐cadherin loss, β‐catenin accumulation and fibronectin expression compared to areas poor in macrophages (Bonde *et al*., 2012). Similarly, such a spatial clustering has been revealed in a human cohort of hepatocellular carcinoma (Fu *et al*., 2015).

**Figure 1:**
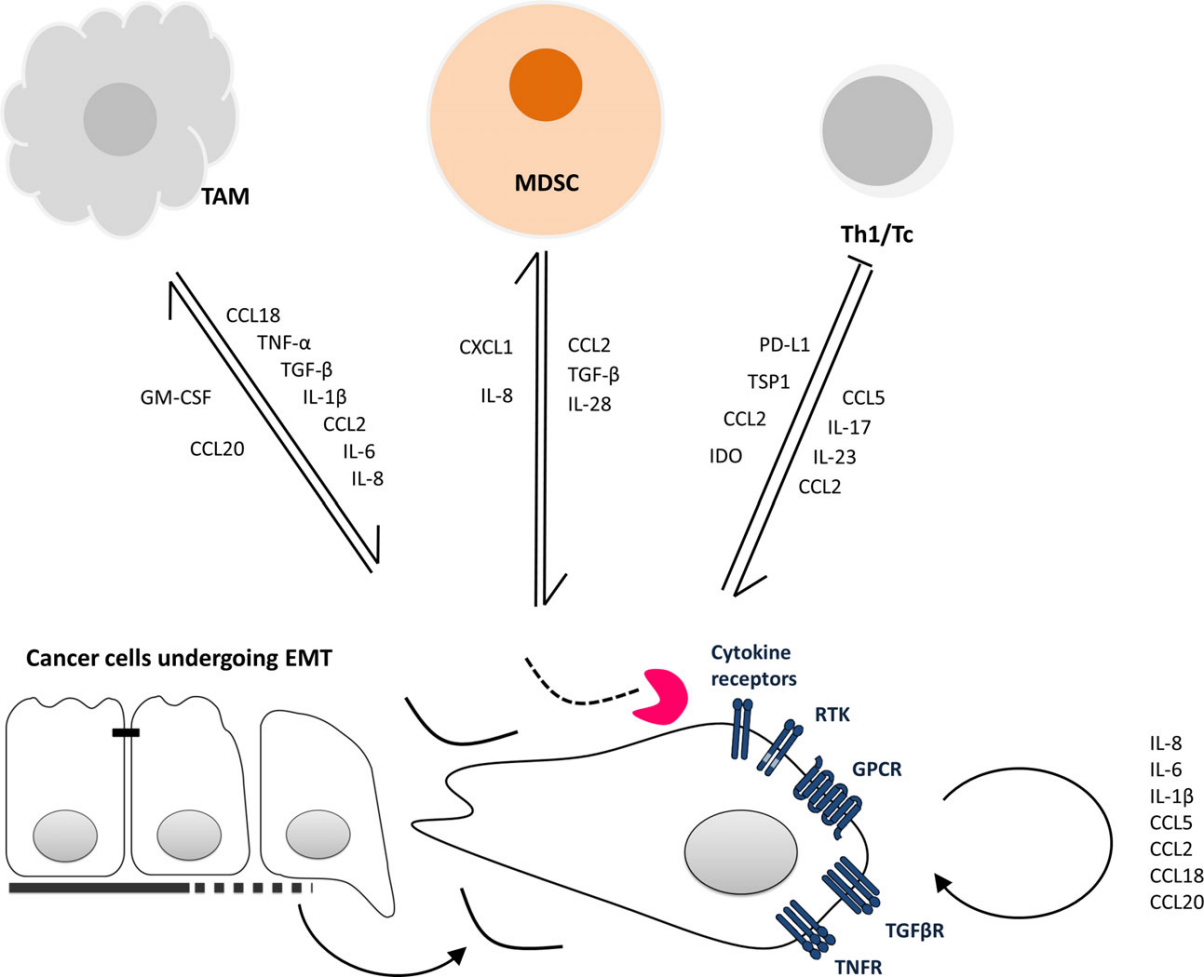
Schematic representation of the soluble factor‐mediated interactions between cancer cells undergoing EMT‐like changes and innate immune cells. RTK, receptor tyrosine kinase; GPCR, G‐protein‐coupled receptor; TAM, tumor‐associated macrophages; MDSCs, myeloid‐derived suppressor cells; Th1/Tc, T helper one cell/cytotoxic T cell. The pink Pacman‐shaped symbol represents some metalloproteinase degrading the extracellular matrix.

Functionally, TAMs have been described as potent EMT inducers in numerous independent studies. TAMs accordingly produce multiple growth factors (HGF, EGF, TGF, PDGF, etc.) and inflammatory cytokines (IL‐1β, IL‐6, and TNF‐α) that each can induce EMT in cancer cells (Fig. 1). *In vitro* data from pancreatic cancer cell lines have demonstrated that co‐culture of cancer cells with M2‐polarized macrophages is able to foster the acquisition of EMT‐like properties in cancer cells, including spindle‐shaped morphology, decreased E‐cadherin and increased vimentin expression, invasive properties, and enhanced production of MMPs (Liu *et al*., 2013). Mechanistically, the TLR4/IL‐10 axis has been involved in this process and inhibition of either effectively represses EMT induction. M2‐TAM‐induced EMT has also been reported in co‐cultures of mouse cancer cells in a murine model of terato‐carcinoma. Interestingly, depletion of macrophages using clodronate liposomes in these mice decreases the expression of mesenchymal features in primary tumors (Bonde *et al*., 2012).

In hepatocellular carcinoma, macrophages induce EMT in cancer cells in co‐culture experiments, in an IL‐8‐dependent fashion (Fu *et al*., 2015) or in a TGF‐β‐dependent fashion (Deng *et al*., 2016; Fan *et al*., 2014). In other tumor types such as NSCLC, macrophages are known to induce EMT via EGF (Ravi *et al*., 2016).

On the other hand, the EMT program in cancer cells might facilitate polarization of TAM into M2‐like, tumor‐supporting cells. Indeed, conditioned media from mesenchymal‐like breast cancer cell lines activate macrophages into a TAM‐like phenotype, including high expression of CD206 (a scavenger receptor also known as macrophage mannose receptor or MMR) and high secretion of CCL17, CCL18, CCL22, and IL‐10 (Su *et al*., 2014). This effect is mediated by the production of GM‐CSF, which has accordingly been associated with EMT in breast cancer (Su *et al*., 2014; Suarez‐Carmona *et al*., 2015). GM‐CSF‐activated macrophages in turn induce EMT in breast cancer cells, in a CCL18‐dependent mechanism (Su *et al*., 2014), establishing a potential local feedback regulation (Fig. 1).

#### 2.1.2 Other myeloid cells

In a transgenic murine model of melanoma (the RETAAD mice, expressing the activated RET oncogene), MDSCs preferentially infiltrated the primary tumor in comparison with metastases. Mostly recruited following a CXCL5 gradient (whose human orthologues, CXCL1 and IL‐8, are expressed by cancer cells), these immunosuppressive cells could in turn promote EMT‐like changes in cancer cells, via multiple pathways comprising TGF, EGF, and HGF (Fig. 1). Most interestingly, depletion of MDSCs using an anti‐Ly6G antibody led to a decrease in EMT marker expression (namely vimentin and S100A4/FSP1) (Toh *et al*., 2011).

A couple of years ago, we observed that conditioned media from cancer cells induced for EMT could recruit CD11b+ GR1+ myeloid cells *in vivo* in a murine model. Similarly, in a cohort of 40 triple‐negative breast cancer samples, tumors infiltrated by high numbers of CD33‐positive myeloid cells were more susceptible to display EMT features (as measured by vimentin expression in above 10% of the tumor cells) (Suarez‐Carmona *et al*., 2015). Accordingly, in a cohort of 97 high‐grade breast cancer patients, a high extracellular matrix‐related gene expression profile (‘ECM3’, characterized by high SPARC and collagen expression) correlates with a high expression of EMT transcription factor (EMT‐TF) Twist1 and high numbers of infiltrating CD33‐positive myeloid cells (Sangaletti *et al*., 2016). Whether these myeloid cells are mature (neutrophils) or immature (MDSCs) cells is still unclear, because of a marker overlap both in mice (CD11b‐Ly6G stain neutrophils and their progenitors) and in humans (CD33 stains the whole myeloid lineage) (Ugel *et al*., 2015). A way to discriminate between immature myeloid cells and neutrophils in human immunohistochemistry studies could be to use a stain for myeloperoxidase (MPO), used in routine to detect finally activated neutrophils, or other markers of differentiated neutrophils, such as CD66b. We mention hereafter two studies in which specifically mature neutrophils were involved in the acquisition of EMT traits by cancer cells. In the first study, the density of infiltrating neutrophils was studied in pancreatic cancer biopsies using a staining for elastase, presumably expressed by differentiated cells (Grosse‐Steffen *et al*., 2012). As a result, high density of neutrophils was related to the nuclear accumulation of beta‐catenin and ZEB1 in tumors. These findings were not reproducible in hepatocellular carcinoma. In addition, co‐culture of neutrophils induced EMT in cancer cells *in vitro*. The mechanism involved was a disruption of E‐cadherin‐mediated cell–cell adhesion by neutrophil‐derived elastase (Grosse‐Steffen *et al*., 2012). In another study, neutrophils were detected using CD66b in lung adenocarcinoma specimens. CD66b is expressed by activated neutrophils and eosinophils. The density of intratumoral CD66b was inversely correlated with E‐cadherin expression (Hu *et al*., 2015). Consistently with the study mentioned above (Grosse‐Steffen *et al*., 2012), co‐culture between neutrophils and lung cancer cell lines resulted in EMT trait acquisition and increased migration, in a process requiring TGF‐β this time (Hu *et al*., 2015).

The important functional role of myeloid cells, particularly immunosuppressive immature myeloid cells (MDSCs), in inducing EMT has been illustrated in an elegant study using high‐grade breast cancers (Sangaletti *et al*., 2016). Ectopic expression of SPARC in high‐grade breast cancer cells leads to the acquisition of EMT features *in vivo* but not *in vitro*, highlighting the role of the microenvironment in this process. Tumors formed by cancer cells ectopically expressing SPARC, in addition to displaying EMT‐like features, produce more metastases and are infiltrated by many myeloid cells (CD11b+GR1+) with strong immunosuppressive functions (as measured by their ability to inhibit anti‐CD3 and anti‐CD28‐induced T‐cell proliferation *in vitro*). Inhibition of these suppressive functions using zoledronic acid effectively reverses EMT in tumors *in vivo*. Moreover, zoledronic acid‐treated, SPARC‐expressing tumor cells have a higher mitotic index and are accordingly more sensitive to Doxil, compared to their untreated counterparts (Sangaletti *et al*., 2016). Supportively, an independent, veterinary group has reported induction of EMT‐like changes *in vitro* by co‐culturing canine tumor cells with MDSCs. This occurs in an IL‐28/IL‐28R‐dependent way (Mucha *et al*., 2014) (Fig. 1).

### 2.2 EMT and adaptive immune cells

Beyond MDSCs, there is a strong connection between EMT and chronic inflammation‐associated immunosuppression in cancer progression (Chen *et al*., 2014, 2015a). This immunosuppression involves regulatory dendritic cells (DCregs), regulatory T cells (Tregs), and effector T‐cell exhaustion by the expression of immune checkpoints.

In mice bearing NSCLC, the immune checkpoint PD‐L1 is detected on tumor cells expressing the EMT‐TF ZEB1. PD‐L1 binds its receptor PD‐1 at the surface of T cells and this is associated with T‐cell dysfunction, decreased tumor‐infiltrating lymphocyte (TIL) density, and metastasis outburst (Chen *et al*., 2014). This link between EMT and immunosuppression seems to be conserved in humans as the authors could identify, using two independent gene expression data sets, that EMT gene signature in lung adenocarcinoma is related to the upregulated expression of multiple immune checkpoints (PD‐1/PD‐L1, CTLA‐A, TIM‐3, and others), to high Foxp3‐positive Treg cell density, to immunosuppressive cytokine production (TGF‐β, IL‐10, and IL‐6), and finally, to a strong inflammatory reaction (indeed, CD4+ Treg cells produce high amounts of proinflammatory cytokines such as IL‐10, TNF‐α, or IL‐6) (Chen *et al*., 2014). This has been recently confirmed in human cancer. Breast cancer cells undergoing EMT indeed express PD‐L1 in a miR‐200‐ and ZEB1‐dependent fashion (Noman *et al*., 2017).

In mouse melanoma xenografts, Snail1 transfectants (which promote Treg expansion *in vitro)* recruit both Tregs and DCregs *in vivo*. The latter would depend on melanoma cell‐derived CCL2 (Kudo‐Saito *et al*., 2013). Local immunosuppression and resistance to DC immunotherapy is suppressed by intratumoral injection of an anti‐Snail1 siRNA, or by anti‐TSP1 (thrombospondin 1) treatment (Kudo‐Saito *et al*., 2009), underlining the central role of EMT programs in cancer progression in this model.

Indoleamine 2,3 dioxygenase (IDO) is mainly a tumor cell‐derived, inflammation‐induced, immunosuppressive factor (Muller *et al*., 2008; Uyttenhove *et al*., 2003) that promotes tumor progression. Ricciardi *et al*. (2015) have recently reported a decreased viability and/or proliferation of natural killer (NK) cells and of T cells after co‐culture with cancer cell lines in which EMT had been induced. This effect was triggered by an IFN‐γ‐mediated IDO overexpression in cancer cells and was blocked by co‐culture in the presence of the IDO inhibitor L‐1MT (Ricciardi *et al*., 2015) (Fig. 1).

Finally, in hepatocellular carcinoma cell lines, hypoxia or HIF‐1α expression triggers EMT‐like changes including E‐cadherin loss and morphological modifications. Simultaneously, HIF‐1α binds to a HRE (hypoxia response element) on the CCL20 promoter and induces CCL20 expression by cancer cells (Ye *et al*., 2016). Hypoxic tumor cell‐derived CCL20 induced IDO expression in macrophages, which leads to decreased T‐cell proliferation and increased Foxp3+ Treg cell proportions after co‐culture (Ye *et al*., 2016).

## 3 EMT and soluble factors of inflammation

Further supporting the relationship between the cellular actors of inflammation and EMT detailed above, extensive literature has also linked EMT and soluble mediators of inflammation. As we review hereafter, several of these soluble mediators, mostly cytokines and chemokines, have been shown to trigger EMT in various cancer cell types. Conversely, EMT‐derived tumor cells have also been shown to overproduce many proinflammatory mediators, thereby establishing a regulatory loop that may contribute to maintain both the EMT phenotype and the proinflammatory context.

It is important to consider that tumor‐associated EMTs rarely involve a complete lineage switching but rather generate intermediate states (hybrid phenotypes) with varying degrees of aggressiveness that distribute along the epithelial‐to‐mesenchymal differentiation spectrum. Correspondingly, there is a multiplicity of EMT molecular repertoires involved in the generation of these hybrid phenotypes. As detailed here under and summarized in Table 1, the extent of EMT induction by specific proinflammatory mediators and the molecular EMT actors affected accordingly vary greatly. If specific responses may be triggered by specific proinflammatory mediators, several EMT‐related changes are commonly induced by several mediators. These common modifications include – but are not limited to – a diminution of E‐cadherin, an induction of vimentin and other mesenchymal markers (FSP1, N‐cadherin, MMP9), an expression of EMT‐TFs, the acquisition of enhanced migratory and invasive properties.

Below, we review *in vitro*‐based studies that identified relationships and regulatory loops linking EMT and specific well‐known proinflammatory mediators. Animal and clinical studies that functionally involve these EMT/cytokine regulatory loops in the metastatic progression are discussed in a separate chapter (Section 4).

### 3.1 TGF‐β

TGF‐β is a key factor of cancer‐related inflammation, but its effects on immune cells are very broad. TGF‐β promotes chemotaxis of eosinophils, macrophages, and mast cells. It skews macrophage and neutrophil polarization into tumor‐promoting, immunosuppressive cells. TGF‐β also negatively impacts the antitumor immune response by multiple mechanisms (inhibition of antigen‐presenting functions of DCs, suppression of cytotoxic functions of CD8+ T cells, promotion of development of inflammatory CD4 T cells such as Th17 or Th9). This has been recently reviewed (Chen and ten Dijke, 2016). Finally, in the tumor microenvironment, production of TGF‐β originates from multiple cells such as tumor cells, fibroblasts, macrophages, leukocytes, endothelial cells (Papageorgis and Stylianopoulos, 2015). The role of TGF‐β in cancer metastasis in general, and in cancer EMT in particular, has been massively documented (for a few examples, see Table 1). It has been reviewed, several times, elsewhere (Moustakas and Heldin, 2016; Papageorgis and Stylianopoulos, 2015; Pickup *et al*., 2013, 2017; Zhang *et al*., 2016a).

### 3.2 TNF‐α

Tumor necrosis factor alpha (TNF‐α) was historically so named because it induces hemorrhagic necrosis of tumors. Its dual role has since been studied and it is now widely accepted that chronic exposure to TNF‐α rather promotes tumor cell proliferation, angiogenesis, and dissemination. TNF‐α can control the expression of multiple other cytokines and TNF‐neutralizing agents are used to treat cancer (Crusz and Balkwill, 2015).

**Figure 2:**
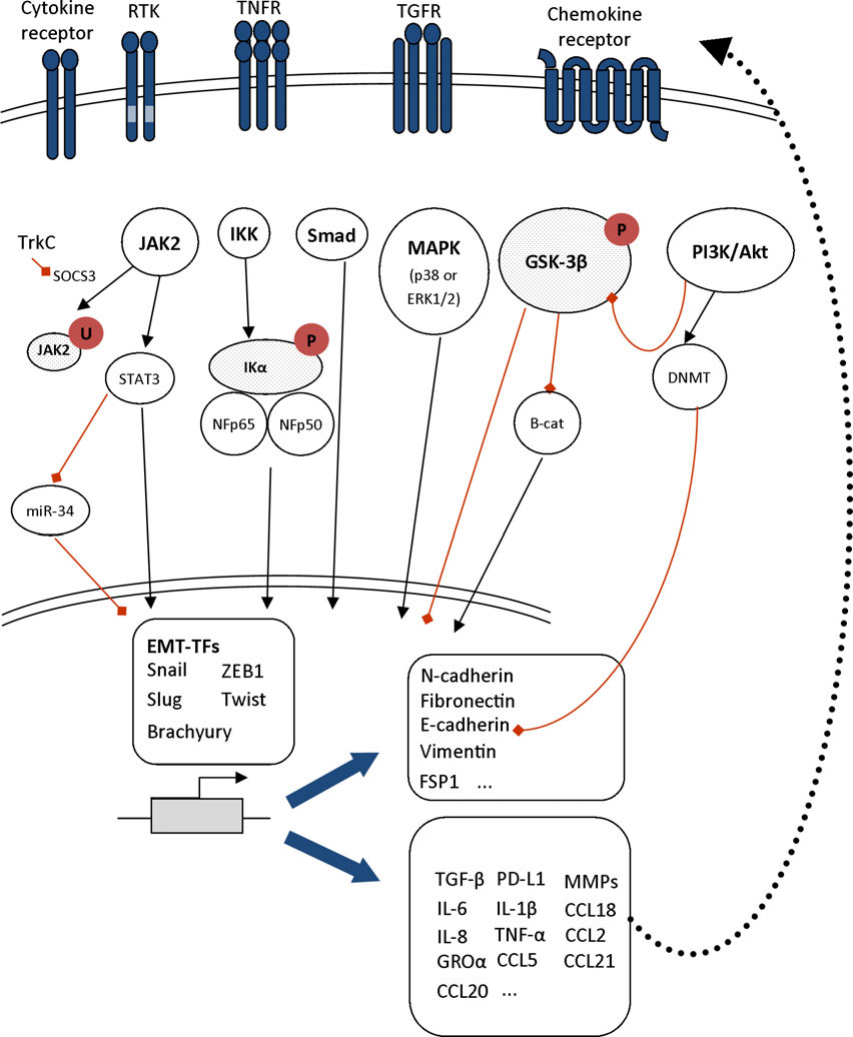
Overview of major signaling cascades leading to EMT program activation and inflammatory target gene activation in cancer cells.

The induction of EMT by TNF‐α, particularly in synergy with TGF‐β or other inflammatory factors, has been described (Bates and Mercurio, 2003) (Figure 2 and Table 1). For instance, a mix of TGF‐β, IFN‐α, and TNF‐α can be used to induce EMT‐like changes in human cancer cell lines *in vitro* (Ricciardi *et al*., 2015). Treatment of cancer cells with the supernatant from a mixed lymphocyte reaction (MLR) experiment induces a similar phenotype (Ricciardi *et al*., 2015). In colorectal cancer cell lines, TNF‐α and TGF‐β induce EMT‐like changes either in a NLRP3/Snail1 axis‐dependent way (NLRP3 stands for ‘NOD‐like receptor family, pyrin domain containing 3’) (Song *et al*., 2016), or via an increase in expression of claudin‐1. In this case, claudin‐1 is delocalized away from the membrane and activates the Src and ERK1/2 MAP kinase pathways (Bhat *et al*., 2016). TNF‐α‐induced EMT has been reported in cell lines from multiple other cancer entities: hepatocellular carcinoma (Zhu *et al*., 2016), breast cancer (Cohen *et al*., 2015), lung cancer (Song *et al*., 2016), or thyroid cancer (here in combination with IFN‐γ) (Lv *et al*., 2015) among others. Interestingly, TNF‐α treatment‐induced EMT signaling pathways in renal cell carcinoma cell lines require expression of CXCR2, CXCR3, and their ligands (Sun *et al*., 2016), highlighting synergy with chemokines.

### 3.3 IL‐1β

Interleukin‐1β (IL‐1β) is a major cytokine involved in cancer‐associated chronic inflammation (Crusz and Balkwill, 2015). A few reports have illustrated a causative link between the IL‐1β/IL‐1R axis and induction of EMT‐like changes in (pre)cancerous cells *in vitro* (Table 1). Li *et al*. (2012b) initially showed that IL‐1β treatment promotes EMT in primary colorectal cancer cells via ZEB1 overexpression. IL‐1β also induces EMT in oral squamous cell carcinoma cell lines and in primary dysplastic oral keratinocytes. Interestingly, in these cells, IL‐1β‐induced EMT leads to enhanced secretion of the proinflammatory cytokines IL‐6, IL‐8, and GROα (Lee *et al*., 2015), thereby initiating a potential autocrine EMT maintenance loop. Finally, in the MCF10A cells, ectopic expression of EMT‐TF Snail1 leads to hyper‐responsiveness to IL‐1β, characterized by high activation of NF‐kB and MAPK pathways and production of IL‐6 and IL‐8 (Lim *et al*., 2013) (Fig. 2). This is in line with the textbook signaling pathway of IL‐1β, which, upon binding to its IL‐1R (a dimeric cytokine receptor), leads to activation of the NF‐κB pathway and initiates a proinflammatory program in a multitude of cell types.

### 3.4 IL‐8

Although IL‐8 can be secreted by a variety of stromal cells, EMT pathways induce IL‐8 expression and secretion in tumor cells as shown in human breast cancer cell lines (Brysse *et al*., 2012; Li *et al*., 2012a; Lim *et al*., 2013; Suarez‐Carmona *et al*., 2015), in immortalized oral keratinocytes (Lyons *et al*., 2008), in lung cancer cell lines (Fernando *et al*., 2011), and in colorectal cancer cell lines (Bates *et al*., 2004) or in colorectal cancer cells freshly isolated from human tumors (Hwang *et al*., 2011). In this last model, Snail1 has been shown to directly promote IL‐8 transcription upon binding to E3/E4 E‐boxes. Finally, there is a correlation between Snail1 and IL‐8 protein expression in a cohort of 52 human colorectal cancer specimens (Hwang *et al*., 2011). Other reports have highlighted the functional role of EMT‐TFs Twist (Li *et al*., 2012a), Brachyury (Fernando *et al*., 2011), and the Snail family (Suarez‐Carmona *et al*., 2015) in the regulation of IL‐8 expression in cancer cell lines. Most recently, Lesage *et al*. (2017) have demonstrated that the molecule ZO‐1, located at the membrane in epithelial cells but transported into the nuclear/cytoplasmic compartment upon EMT‐associated junction disruption, activates the secretion of IL‐8 via the NF‐kB pathway in breast and lung cancer cell lines.

Conversely, IL‐8 can induce EMT (Table 1). IL‐8 can bind to two G‐protein‐coupled receptors, CXCR1/2, and initiate several signaling pathways, such as Ras/Raf/MAPK, PI3K, or JAK/STAT (Fig. 2). IL‐8‐mediated EMT has been demonstrated in ovarian cancer cell lines (Yin *et al*., 2015), breast (Ji *et al*., 2016), and lung cancer cells. In these, erlotinib‐induced autocrine IL‐8 production induces EMT via the p38 MAPK kinase pathway (Fig. 2). IL‐8 neutralization effectively restores an epithelial phenotype (Fernando *et al*., 2016). IL‐8 additionally triggers EMT changes in thyroid cancer cells via the AKT/Slug pathway (Visciano *et al*., 2015) and in hepatocellular carcinoma, via JAK2/STAT3 activation and Snail1 expression (Fu *et al*., 2015). Finally, Zhang *et al*. (2016e) have recently shown that, in nasopharyngeal carcinoma cells, IL‐8 stabilizes the DN methyl‐transferase DNMT1 via AKT, thereby inducing methylation of E‐cadherin promoter, leading to E‐cadherin repression and EMT program activation. Cooperative induction of EMT in colorectal cancer cells has been shown upon IL‐8 and CCL20 treatment, in conditions in which each individual treatment failed to induce EMT. This combined effect involves the activation of the PI3K, Akt, and ERK1/2 pathways (Cheng *et al*., 2014).

Altogether, the data linking IL‐8 and EMT have established the principle of a mutual loop in which IL‐8 and EMT programs sustain each other in the cancer microenvironment (Long *et al*., 2016).

### 3.5 IL‐6

The role of IL‐6 in cancer‐related inflammation has been reviewed elsewhere (Pesic and Greten, 2016). IL‐6, produced both by tumor cells (especially after Ras or p53 mutations) and by reactive stromal cells (Crusz and Balkwill, 2015), promotes both local and systemic inflammation. Elevated circulating levels of IL‐6 are an indicator of a poor outcome in several cancer entities (Crusz and Balkwill, 2015). IL‐6 binds to a dimeric cytokine receptor composed of IL‐6Rα and IL‐6Rβ/gp130. The full receptor is expressed solely by immune cells, while gp130 expression is ubiquitous. In tumors, tumor cells can produce a soluble form of IL‐6Rα which binds to IL‐6, enabling the so‐formed dimer to interact with gp130 at the surface of cancer cells and initiate prosurvival signaling (Naugler and Karin, 2008).

The reciprocal causative link between EMT pathways and IL‐6 has been supported by a number of independent studies (Table 1). Historically, the negative effect of IL‐6 on E‐cadherin expression in breast cancer cell lines was already demonstrated almost 20 years ago (Asgeirsson *et al*., 1998). Later, Sullivan *et al*. (2009) demonstrated the induction of EMT phenotypes in estrogen receptor alpha‐positive breast cancer cells. In this work, the ectopic expression of IL‐6 in MCF7 cells activated the JAK2/STAT3 pathway and Twist, leading to the acquisition of mesenchymal markers and properties (Fig. 2). More recently, a role for IL‐6 in inducing and maintaining EMT was shown in other breast cancer cell lines. IL‐6, induced by TrkC (tropomyosin receptor kinase C), triggers EMT and Twist1‐mediated IL‐6 overexpression, initiating a vicious loop securing the EMT phenotype (Kim *et al*., 2016). IL‐6 also induces EMT *in vitro* in immortalized epithelial oral cells, in a JAK2/STAT3/Snail1‐dependent way (Yadav *et al*., 2011), in lung adenocarcinoma cells (in a STAT3‐Snail1‐dependent fashion) (Zhao *et al*., 2014) and in NSCLC cells (Lee *et al*., 2016; Shintani *et al*., 2016). In this latter case, the effects of IL‐6 are more pronounced in the CD133+, stem‐like, subpopulation (Lee *et al*., 2016). IL‐6 also induces EMT in ovarian cancer cells, similarly via the JAK2/STAT3 signaling pathway (Colomiere *et al*., 2009) (Fig. 2), in head and neck squamous cell carcinoma cells (Wu *et al*., 2016a), in gastric cancer cells (Chen *et al*., 2017), and in uterine cervix cancer cells (in a STAT3‐Slug‐dependent way) (Miao *et al*., 2014). Finally, an additional pathway has been linked to IL‐6‐mediated EMT in colorectal cancer cells (Rokavec *et al*., 2014). Phosphorylated STAT3 can directly repress miR‐34 (a miRNA involved in the maintenance of the epithelial phenotype, mostly by inhibiting the EMT‐TF Snail1) by binding on its first intron. miR‐34 is a p53‐regulated target that represses IL‐6Rα (Rokavec *et al*., 2014).

On the contrary, the induction of EMT in cancer cell lines can foster expression of IL‐6 at quite high levels in breast cancer cell lines via Twist (Sullivan *et al*., 2009) or Snail1 (Suarez‐Carmona *et al*., 2015) and in oral keratinocytes via Snail1 (Lyons *et al*., 2008). The pathways involved in Snail1‐mediated IL‐6 (and IL‐8) mRNA expression have been detailed in the immortalized breast cells MCF10A (Lim *et al*., 2013) and comprise ERK, STAT2/3, and AKT activation.

### 3.6 Th17‐related cytokines

Tumor infiltration by the proinflammatory Th17 T cells is associated with increased Th17‐associated cytokines IL‐23, IL‐17, IL‐1β, and IL‐6. In esophageal cancer, IL‐23 expression in the primary tumor relates to the presence of nodal and distance metastases (Chen *et al*., 2015). *In vitro*, treatment of esophageal cancer cell lines with recombinant IL‐23 induces EMT‐like changes characterized by loss of E‐cadherin and *de novo* vimentin expression (Chen *et al*., 2015). The signaling pathways in these cells comprise activation of the Wnt/β‐catenin pathway, subsequent GSK‐3β stabilization, which finally triggers Slug and Snail1 expression (Fig. 2). Regarding IL‐17, treatment with recombinant IL‐17 was shown to induce EMT‐like changes in three human cell lines (Zhang *et al*., 2016d) (Table 1). Similarly, treatment of quiescent gastric cancer stem cells with IL‐17 also results in EMT‐like changes including E‐cadherin loss, *de novo* vimentin acquisition, and increased migratory and invasive properties (Jiang *et al*., 2017) and associates with STAT3 phosphorylation.

### 3.7 CCL2

CCL2 [or monocyte chemotactic protein 1 (MCP1)] is produced by several cell types and recruits most immune cells (except from neutrophils and eosinophil) by binding to its receptor CCR2. CCL2 expression in cancer correlates with TAM density (Deshmane *et al*., 2009).

CCL2 expression leads to EMT in several cancer cell types (Table 1). In lung cancer cell lines, CCL2 and IL‐6 synergistically induce EMT in a Twist/STAT3‐dependent fashion (Chen *et al*., 2015b) (Fig. 2). CCL2 neutralization also inhibits EMT in prostate cancer cells (Izumi *et al*., 2013). Finally, in bladder cancer, mast cells induce EMT in cancer cells via stimulation of the ERβ/CCL2/CCR2 axis (Rao *et al*., 2016).

On the contrary, in mouse melanoma cells, ectopic Snail1 expression leads to EMT and subsequently to CCL2 production (Kudo‐Saito *et al*., 2013).

### 3.8 CCL5

CCL5 is a proinflammatory chemokine involved in inflammatory cell recruitment, cancer‐related angiogenesis, and tumor metastasis formation (Aldinucci and Colombatti, 2014). Its receptors, CCR1, CCR3, and mostly CCR5, are expressed at the surface of myeloid cells, T cells, and tumor cells. Recently, CCL5 has emerged as an EMT inducer (Table 1). CCL5 was indeed identified as a T cell‐derived, pro‐EMT factor in human colorectal cancer liver metastasis tissue (Halama *et al*., 2016). In ovarian cancer models, CCL5 has also been shown to be produced by CD133‐positive stem‐like cancer cells. By binding to CCR1/3/5 on CD133‐negative, non‐stem‐like cells, CCL5 activates NF‐κB and thereby promotes EMT and metastasis (Long *et al*., 2015). Finally, CCL5 derived from the adipose microenvironment fosters invasive properties of triple‐negative breast cancer cells *in vitro* (D'Esposito *et al*., 2016).

### 3.9 CCL18

CCL18 is a macrophage‐derived, T cell‐attracting chemokine. CCL18 is only expressed in human, not in mice; recent data indicate that it binds to CCR8 (Islam *et al*., 2013).

A few reports have linked CCL18 to EMT in cancer (Table 1). In pancreatic cancer cells for example, CCL18 is expressed both by tumor cells and by M2‐polarized macrophages. *In vitro*, recombinant CCL18 induces migration, and invasion, as well as EMT‐TF Snail1 expression in pancreatic cancer cell lines (Meng *et al*., 2015). In breast cancer cell lines, CCL18 derived from TAMs activates NF‐κB and thereby induces EMT‐like changes including spindle‐shaped morphology, E‐cadherin‐to‐vimentin switch, and increased invasive properties (Su *et al*., 2014).

Finally in the ovarian cancer cell line Skov3, CCL18 ectopic expression endows tumor cells with enhanced migratory and invasive properties *in vitro* (although the authors do not investigate the expression of any marker or transcription factor linked to EMT) (Wang *et al*., 2016).

### 3.10 CCL20

In human colorectal cancer, CCL20 receptor CCR6 is overexpressed in tumors compared to normal tissue, and expression intensity increases with tumor stage, suggesting a functional role in aggressiveness. Supportively, the authors also show that colorectal cancer cells undergo EMT changes when exposed to CCL20 *in vitro*. This is inhibited by neutralizing CCR6 (Kapur *et al*., 2016) (Table 1). Additionally, co‐occurrence of CCL20 expression and EMT has been reported in hepatocellular cancer (Ye *et al*., 2016), as described in Section 2.2.

### 3.11 CCL21

In a panel of pancreatic cancer cell lines, CCR7 is found overexpressed specifically in stem‐like CD133‐positive cells (Table 1). Activation of this receptor by CCL21 leads to the acquisition of EMT markers (E‐to‐N‐cadherin switch, MMP9 expression) and increased invasive properties. This involves Erk/NF‐κB signaling (Zhang *et al*., 2016c).

Expression of the CCL21 receptor CCR7 is also increased in breast cancer cells upon TGF‐β‐induced EMT, which facilitates breast cancer cell migration toward CCL21‐positive, draining lymphatic vessels in mice, in a DC‐like fashion (Pang *et al*., 2016).

### 3.12 Reactive oxygen species

During an inflammatory reaction, ROS are mostly produced by activated macrophages or neutrophils. They activate adaptive immune cells and contribute to apoptosis induction. In addition, ROS are major regulators of the production of proinflammatory cytokines and chemokines (such as CXCL12) (Costa *et al*., 2014). Whether these major actors of inflammation impact the EMT program in cancer cells has been investigated for the past few years.

Nevertheless, the effect of ROS on the induction – or inhibition – of EMT programs in cancer cells is unclear, as opposite effects of ROS on EMT induction in cancer cells have been described. The production of ROS has been shown to inhibit EMT in two prostate cancer cell lines (Das *et al*., 2014). Similarly, in basal‐like breast carcinoma cells, Snail1‐induced epigenetic silencing of FBP1 (fructose‐1, 6‐bisphosphatase) on the one hand promotes glycolysis and the production of NAPDH (via the pentose phosphate pathway), and on the other hand decreases respiration, leading to the decreased production of ROS, which finally endows tumor cells with cancer stem cell‐ and EMT‐like features (Dong *et al*., 2013). Conversely, the inhibition of mitochondrial ROS in cervical cancer cells reverses the acquisition of EMT‐like properties (Shagieva *et al*., 2016). In colorectal cancer cell lines, oxaliplatin‐induced EMT depends on ROS production (Jiao *et al*., 2016). Accordingly, the drug nimbolide, which has been successfully used to reduce metastasis in a mouse pancreatic cancer model, inhibits EMT by inducing excessive production of ROS (Subramani *et al*., 2016).

## 4 EMT, inflammation, and the metastatic progression

There has been a plethora of studies consistently linking chronic inflammation to cancer progression and metastatic outgrowth (Crusz and Balkwill, 2015; Grivennikov and Karin, 2010; Muller *et al*., 2008; Naugler and Karin, 2008; Pesic and Greten, 2016). Similarly, many publications have illustrated the prometastatic effects of EMT program activation in cancer cells (Bates and Mercurio, 2005; Fassina *et al*., 2012; Spaderna *et al*., 2006; Yu *et al*., 2013). Research data indicating that an alliance, or mutual loop, between EMT‐like cancer cells and established chronic inflammation promotes metastasis in a cooperative fashion are rather scarce. We here focus on these few studies and mention the role of the EMT/inflammation axis in tumorigenesis, invasion, liberation of circulating tumor cells, and metastasis outburst.

The EMT/inflammation axis is already involved in the very first steps of tumor formation. For instance, ectopic expression of Snail1 in colorectal cancer cells induces properties of colonospheres including stem cell properties and IL‐8 expression, leading to increased tumorigenicity in nude mice (Hwang *et al*., 2011). Similarly, subcutaneous injection of gastric quiescent cancer stem cells to nude mice after 24 h of treatment with IL‐17, which induces EMT‐like changes *in vitro*, results in the formation of larger xenografts in nude mice (Jiang *et al*., 2017). Accordingly, in PTEN^null^ MMP7^null^ mice and PTEN^null^ IL‐17RC^null^ mice, prostate tumors are smaller and a lower proportion of mice develop invasive adenocarcinoma compared to PTEN single‐KO mice. These changes are accompanied by weaker EMT‐like characteristics in cancer cells (Zhang *et al*., 2016d).

Primary tumors can also become resistant to therapy because of the EMT/inflammation alliance. In lung cancer cells, erlotinib‐induced autocrine IL‐8 production induces EMT and triggers erlotinib resistance via the p38 MAP kinase pathway. IL‐8 neutralization effectively restores both an epithelial phenotype and erlotinib sensitivity (Fernando *et al*., 2016).

EMT in association with inflammation has also been connected with higher stages of cancer progression in several studies. An important step in metastatic progression of tumors is the liberation and survival of circulating tumor cells. In patients with inflammatory breast cancer, a correlation exists between immune activation and the presence of circulating tumor cells (CTCs) with EMT characteristics (Cohen *et al*., 2015). Indeed, among 16 tested patients, six patients have high numbers of circulating TNF‐α‐producing T cells. Of these six patients, four had EMT‐like CTCs. In contrast, only one patient with low numbers of TNF‐producing T cells (among 10) has EMT‐like CTCs (Cohen *et al*., 2015).

Finally, most clinical data regarding the EMT/inflammation axis have highlighted its correlation with metastatic stage and poor outcome. This has been proven to be relevant in multiple cancer entities. In colorectal cancer specimens, concomitant expression of IL‐8 and CCL20 correlates with E‐cadherin loss and with lymph node and liver metastasis (Cheng *et al*., 2014). CCL20‐IL‐8 double positivity is also linked to a shorter overall survival (OS) and disease‐free survival (DFS) in these patients. Still in colorectal cancer, the expression of IL‐6 and STAT3 and the absence of miR‐34 (altogether constituting an EMT‐maintaining loop) are associated with nodal and distant metastases in a cohort of 425 patients (Rokavec *et al*., 2014). In breast cancer, the expression of Shohl2 – a transcription factor of the bHLH family which inhibits EMT by repressing IL‐8 expression *in vitro* (Ji *et al*., 2016) – is reduced in metastases compared to primary tumors. In mouse xenografts, ectopic expression of Shohl2 inhibits metastasis outgrowth, while Shohl2 knockdown boosts metastasis formation. Finally, Shohl2 and IL‐8 protein expressions are negatively correlated in a cohort of 12 breast cancer samples (Ji *et al*., 2016). The CCL18/GM‐CSF/EMT loop described above links EMT to macrophages in a prometastatic pathway. CCL18 and GM‐CSF expression are correlated (a) with each other and (b) with EMT‐like features in human breast cancer specimens, and GM‐CSF expression predicts a shorter DFS (Su *et al*., 2014).

In ovarian carcinoma, CCL5 is produced by ovarian cancer stem‐like cells, where it induces EMT in non‐cancer stem‐like cells. CCL5 and CCR5 expressions are also associated with ovarian cancer metastasis (Long *et al*., 2015). CCL18, which induces EMT in ovarian cancer cells *in vitro*, is also associated with high tumor grade and metastasis in patients with ovarian cancer (Wang *et al*., 2016). High EMT/hypoxia‐associated CCL20 expression has been associated with poor patient DFS and OS in a cohort of 90 hepatocellular carcinomas (Ye *et al*., 2016). In two independent cohorts of patients with gastric cancer – in which TAM density is associated with the expression of EMT features in cancer cells – both TAM density and EMT features are related to a shorter cumulative survival (Yan *et al*., 2016; Zhang *et al*., 2016b).

In addition, mouse models have been extensively used to further dissect the EMT/inflammation/metastasis axis. Using a murine model of breast cancer, De Cock *et al*. (2016) have elegantly illustrated the functional synergy between inflammation and cancer in the promotion of metastatic outgrowth. They have generated a dormant cell line that is tumorigenic after orthotopic injection, disseminates in the lung but fails to grow there. These cells express low levels of EMT‐TFs Snail1, Twist1, and ZEB1. Interestingly, doxycycline‐inducible Snail1 or ZEB1 ectopic expression increases metastatic outgrowth. Most importantly, lung inflammation, induced by treating the mice with LPS, also leads to a dramatic increase in metastases formation, which can be effectively inhibited by ZEB1 knockdown. This phenomenon occurs in NOD‐SCID mice and is therefore independent of any adaptive immune response, but intriguingly, LPS‐induced metastatic outgrowth is inhibited by neutrophil depletion. So this indicates that EMT and inflammation are mechanistically required to permit metastasis in this model. In a xenograft model of lung adenocarcinoma, injection of cancer cells that stably overexpress IL‐6 leads to the formation of tumors with EMT characteristics (E‐cadherin loss and vimentin expression) and in an increased number of lung metastases (Zhao *et al*., 2014). In another study, IL‐17‐induced EMT results in increased metastatic numbers in nude mice, in the above‐mentioned gastric cancer xenograft model (Jiang *et al*., 2017). In murine melanoma RETAAD mice presented above, depletion of MDSCs leads to a decrease in EMT marker expression, impairment of tumor growth, and decreased cancer cell dissemination (Toh *et al*., 2011). In melanoma xenograft models, Snail1 transfectants (or cells expressing Snail1 following TGF‐β treatment) acquire EMT features and this results in a shorter overall survival of mice (Kudo‐Saito *et al*., 2009). This aggressiveness relies on local immunosuppression, as characterized by TSP1 expression, impairment of dendritic cells, and recruitment of Tregs.

Finally, mast cells promote metastasis of bladder cancer in mice, in an EMT‐dependent fashion. It has been shown that when mast cells are co‐cultured with bladder cancer cells, CCL2 expression is elevated (after ERβ decrease) and EMT is activated in cancer cells, which express MMP‐9 and display enhanced invasive properties. In mouse xenograft, co‐implantation of mast cells with tumor cells increases metastatic burden. This metastatic advantage is reversed by an ERβ antagonist. So globally, mast cells promote metastasis of bladder cancer in mice in a CCL2/CCR2/EMT/MMP9‐dependent mechanism (Rao *et al*., 2016).

## 5 Concluding remarks

Studying EMT and inflammation has been difficult because both phenomena are highly regulated in time and space. All data collected from immunohistochemistry in human specimens are therefore only snapshots that provide associative data. Furthermore, most available models to study EMT in time lines are either cell‐based models (migration, invasiveness *in vitro*) that fail to reconstitute the complexity in the living environment, or mouse models, in which the study of inflammation, or at least its translation into human data, is complicated by the discrepancies in inflammatory markers between both species.

There is clearly, however, a multifaceted, sustained crosstalk between cancer cells undergoing EMT‐like changes and the cellular actors of inflammation. Tumors are generally described as a wound that never heals, generating a chronic, unresolved inflammatory reaction, which is fueled by a local cytokine storm. EMT programs most certainly play a central role in establishing and maintaining this local cytokine storm. Reciprocally, inflammatory cells present at the tumor site keep producing EMT‐inducing factors. Supportively, tumor cells displaying EMT‐like features are most often encountered at the periphery of tumor islets, at the tumor–stroma interface.

The EMT/inflammation axis definitely fosters aggressiveness of the primary tumors as illustrated in a number of mouse models, and markers of EMT and inflammation are correlated with poor outcome in patient cohorts of multiple cancers. To this day, several anti‐inflammatory agents have been developed and tested for cancer treatment (Crusz and Balkwill, 2015). Conversely, despite a likely implication of EMT in specific steps of the metastatic progression, EMT‐positive tumor cells have not been successfully tackled yet. In this context, we believe that a better understanding of the mechanisms behind the EMT/inflammation alliance might open new perspectives on anti‐inflammatory therapeutic strategies.

## References

[mol212095-bib-0001] Aldinucci D and Colombatti A (2014) The inflammatory chemokine CCL5 and cancer progression. Mediators Inflamm 2014, 292376. 24523569 10.1155/2014/292376 PMC3910068

[mol212095-bib-0002] Asgeirsson KS , Olafsdottir K , Jonasson JG and Ogmundsdottir HM (1998) The effects of IL‐6 on cell adhesion and e‐cadherin expression in breast cancer. Cytokine 10, 720–728. 9770334 10.1006/cyto.1998.0349

[mol212095-bib-0003] Bates RC , DeLeo MJ 3rd and Mercurio AM (2004) The epithelial‐mesenchymal transition of colon carcinoma involves expression of IL‐8 and CXCR‐1‐mediated chemotaxis. Exp Cell Res 299, 315–324. 15350531 10.1016/j.yexcr.2004.05.033

[mol212095-bib-0004] Bates RC and Mercurio AM (2003) Tumor necrosis factor‐alpha stimulates the epithelial‐to‐mesenchymal transition of human colonic organoids. Mol Biol Cell 14, 1790–1800. 12802055 10.1091/mbc.E02-09-0583 PMC165077

[mol212095-bib-0005] Bates RC and Mercurio AM (2005) The epithelial‐mesenchymal transition (EMT) and colorectal cancer progression. Cancer Biol Ther 4, 365–375. 15846061 10.4161/cbt.4.4.1655

[mol212095-bib-0006] Becht E , Giraldo NA , Germain C , de Reynies A , Laurent‐Puig P , Zucman‐Rossi J , Dieu‐Nosjean MC , Sautes‐Fridman C and Fridman WH (2016) Immune contexture, immunoscore, and malignant cell molecular subgroups for prognostic and theranostic classifications of cancers. Adv Immunol 130, 95–190. 26923001 10.1016/bs.ai.2015.12.002

[mol212095-bib-0007] Bhat AA , Ahmad R , Uppada SB , Singh AB and Dhawan P (2016) Claudin‐1 promotes TNF‐α‐induced epithelial‐mesenchymal transition and migration in colorectal adenocarcinoma cells. Exp Cell Res 349, 119–127. 27742576 10.1016/j.yexcr.2016.10.005 PMC6166648

[mol212095-bib-0008] Bonde AK , Tischler V , Kumar S , Soltermann A and Schwendener RA (2012) Intratumoral macrophages contribute to epithelial‐mesenchymal transition in solid tumors. BMC Cancer 12, 35. 22273460 10.1186/1471-2407-12-35 PMC3314544

[mol212095-bib-0009] Brysse A , Mestdagt M , Polette M , Luczka E , Hunziker W , Noel A , Birembaut P , Foidart JM and Gilles C (2012) Regulation of CXCL8/IL‐8 expression by zonula occludens‐1 in human breast cancer cells. Mol Cancer Res 10, 121–132. 22064657 10.1158/1541-7786.MCR-11-0180

[mol212095-bib-0010] Chen W , Gao Q , Han S , Pan F and Fan W (2015b) The CCL2/CCR2 axis enhances IL‐6‐induced epithelial‐mesenchymal transition by cooperatively activating STAT3‐Twist signaling. Tumour Biol 36, 973–981. 25318604 10.1007/s13277-014-2717-z

[mol212095-bib-0011] Chen L , Gibbons DL , Goswami S , Cortez MA , Ahn YH , Byers LA , Zhang X , Yi X , Dwyer D , Lin W *et al* (2014) Metastasis is regulated via microRNA‐200/ZEB1 axis control of tumour cell PD‐L1 expression and intratumoral immunosuppression. Nat Commun 5, 5241. 25348003 10.1038/ncomms6241 PMC4212319

[mol212095-bib-0012] Chen L , Heymach JV , Qin FX‐F and Gibbons DL (2015a) The mutually regulatory loop of epithelial–mesenchymal transition and immunosuppression in cancer progression. OncoImmunology 4, e1002731. 26155392 10.1080/2162402X.2014.1002731 PMC4485725

[mol212095-bib-0300] Chen D , Li W , Liu S , Su Y , Han G , Xu C , Liu H , Zheng T , Zhou Y and Mao C (2015) Interleukin‐23 promotes the epithelialmesenchymal transition of oesophageal carcinoma cells 11 via the Wnt/b‐catenin pathway. Sci Rep 5, 8604. 25721268 10.1038/srep08604 PMC4342574

[mol212095-bib-0013] Chen G , Tang N , Wang C , Xiao L , Yu M , Zhao L , Cai H , Han L , Xie C and Zhang Y (2017) TNF‐α‐inducing protein of *Helicobacter pylori* induces epithelial‐mesenchymal transition (EMT) in gastric cancer cells through activation of IL‐6/STAT3 signaling pathway. Biochem Biophys Res Comm 484, 311–317. 28130110 10.1016/j.bbrc.2017.01.110

[mol212095-bib-0014] Chen W and ten Dijke P (2016) Immunoregulation by members of the TGFβ superfamily. Nat Rev Immunol 16, 723–740. 27885276 10.1038/nri.2016.112

[mol212095-bib-0015] Cheng XS , Li YF , Tan J , Sun B , Xiao YC , Fang XB , Zhang XF , Li Q , Dong JH , Li M *et al* (2014) CCL20 and CXCL8 synergize to promote progression and poor survival outcome in patients with colorectal cancer by collaborative induction of the epithelial‐mesenchymal transition. Cancer Lett 348, 77–87. 24657657 10.1016/j.canlet.2014.03.008

[mol212095-bib-0016] Cohen EN , Gao H , Anfossi S , Mego M , Reddy NG , Debeb B , Giordano A , Tin S , Wu Q , Garza RJ *et al* (2015) Inflammation mediated metastasis: immune induced epithelial‐to‐mesenchymal transition in inflammatory breast cancer cells. PLoS One 10, e0132710. 26207636 10.1371/journal.pone.0132710 PMC4514595

[mol212095-bib-0017] Colomiere M , Ward AC , Riley C , Trenerry MK , Cameron‐Smith D , Findlay J , Ackland L and Ahmed N (2009) Cross talk of signals between EGFR and IL‐6R through JAK2/STAT3 mediate epithelial‐mesenchymal transition in ovarian carcinomas. Br J Cancer 100, 134–144. 19088723 10.1038/sj.bjc.6604794 PMC2634691

[mol212095-bib-0018] Costa A , Scholer‐Dahirel A and Mechta‐Grigoriou A (2014) The role of reactive oxygen species and metabolism on cancer cells and their microenvironment. Semin Cancer Biol 25, 23–32. 24406211 10.1016/j.semcancer.2013.12.007

[mol212095-bib-0019] Crusz SM and Balkwill FR (2015) Inflammation and cancer: advances and new agents. Nat Rev Clin Oncol 12, 584–596. 26122183 10.1038/nrclinonc.2015.105

[mol212095-bib-0020] Das TP , Suman S and Damodaran C (2014) Induction of reactive oxygen species generation inhibits epithelial–mesenchymal transition and promotes growth arrest in prostate cancer cells. Mol Carcinog 53, 537–547. 23475579 10.1002/mc.22014 PMC3795941

[mol212095-bib-0021] De Cock J , Shibue T , Dongre A , Keckesova Z , Reinhardt F and Weinberg RA (2016) Inflammation triggers Zeb1‐dependent escape from tumor latency. Cancer Res 76, 6778–6784. 27530323 10.1158/0008-5472.CAN-16-0608 PMC5135644

[mol212095-bib-0022] Deng Y‐R , Liu W‐B , Lian Z‐X , Li X and Hou X (2016) Sorafenib inhibits macrophage‐mediated epithelial‐mesenchymal transition in hepatocellular carcinoma. Oncotarget 7, 38292–38305. 27203677 10.18632/oncotarget.9438 PMC5122390

[mol212095-bib-0023] Deshmane SL , Kremlev S , Amini S and Sawaya BE (2009) Monocyte chemoattractant protein‐1 (MCP‐1): an overview. J Interferon Cytokine Res 29, 313–326. 19441883 10.1089/jir.2008.0027 PMC2755091

[mol212095-bib-0024] D'Esposito V , Liguoro D , Abrosio MR , Collina F , Cantile M , Spinelli R , Raciti GA , Miele C , Valentino R , Campiglia P *et al* (2016) Adipose microenvironment promotes triple negative breast cancer cell invasiveness and dissemination by producing CCL5. Oncotarget 7, 24495–24509. 27027351 10.18632/oncotarget.8336 PMC5029717

[mol212095-bib-0026] Diepenbruck M and Christofori G (2016) Epithelial–mesenchymal transition (EMT) and metastasis: yes, no, maybe? Curr Opin Cell Biol 43, 7–13. 27371787 10.1016/j.ceb.2016.06.002

[mol212095-bib-0027] Dong C , Yuan T , Wu Y , Wang Y , Fan TW , Miriyala S , Lin Y , Yao J , Shi J , Kang T *et al* (2013) Loss of FBP1 by Snail‐mediated repression provides metabolic advantages in basal‐like breast cancer. Cancer Cell 23, 316–331. 23453623 10.1016/j.ccr.2013.01.022 PMC3703516

[mol212095-bib-0028] Elghonaimy EA , Ibrahim SA , Youns A , Hussein Z , Nouh MA , El‐Mamlouk T , El‐Shinawi M and Mostafa Mohamed M (2016) Secretome of tumor‐associated leukocytes augment epithelial‐mesenchymal transition in positive lymph node breast cancer patients via activation of EGFR/Tyr845 and NF‐kappaB/p65 signaling pathway. Tumour Biol 37, 12441–12453. 27329104 10.1007/s13277-016-5123-x

[mol212095-bib-0029] Fan Q‐M , Jing Y‐Y , Yu G‐F , Kou X‐R , Ye F , Gao L , Li R , Zhao Q‐D , Yang Y , Lu Z‐H *et al* (2014) Tumor‐associated macrophages promote cancer stem cell‐like properties via transforming growth factor‐beta1‐induced epithelial–mesenchymal transition in hepatocellular carcinoma. Cancer Lett 352, 160–168. 24892648 10.1016/j.canlet.2014.05.008

[mol212095-bib-0030] Fassina A , Cappellesso R , Guzzardo V , Dalla Via L , Piccolo S , Ventura L and Fassan M (2012) Epithelial‐mesenchymal transition in malignant mesothelioma. Modern Pathol 25, 86–99. 10.1038/modpathol.2011.144 21983934

[mol212095-bib-0031] Fernando RI , Castillo MD , Litzinger M , Hamilton DH and Palena C (2011) IL‐8 signaling plays a critical role in the epithelial‐mesenchymal transition of human carcinoma cells. Can Res 71, 5296–5306. 10.1158/0008-5472.CAN-11-0156 PMC3148346 21653678

[mol212095-bib-0032] Fernando RI , Hamilton DH , Dominguez C , David JM , McCampbell KK and Palena C (2016) IL‐8 signaling is involved in resistance of lung carcinoma cells to erlotinib. Oncotarget 7, 42031–42044. 27248176 10.18632/oncotarget.9662 PMC5173114

[mol212095-bib-0033] Fischer KR , Durrans A , Lee S , Sheng J , Li F , Wong ST , Choi H , El Rayes T , Ryu S , Troeger J *et al* (2015) Epithelial‐to‐mesenchymal transition is not required for lung metastasis but contributes to chemoresistance. Nature 527, 472–476. 26560033 10.1038/nature15748 PMC4662610

[mol212095-bib-0034] Fu X‐T , Song K , Zhang Z‐J , Zhou Z‐J , Zhou S‐L , Zhao Y‐M , Xiao Y‐S , Sun Q‐M , Ding Z‐B and Fan J (2015) Macrophage‐secreted IL‐8 induces epithelial‐mesenchymal transition in hepatocellular carcinoma cells by activating the JAK2/STAT3/Snail pathway. Int J Oncol 46, 587–596. 25405790 10.3892/ijo.2014.2761

[mol212095-bib-0035] Grivennikov SI and Karin M (2010) Inflammation and oncogenesis: a vicious connection. Curr Opin Genet Dev 20, 65–71. 20036794 10.1016/j.gde.2009.11.004 PMC2821983

[mol212095-bib-0036] Grosse‐Steffen T , Giese T , Giese N , Longerich T , Schirmacher P , Haensch GM and Gaida MM (2012) Epithelial‐to‐mesenchymal transition in pancreatic ductal adenocarcinoma and pancreatic tumor cell lines: the role of neutrophils and neutrophil‐derived elastase. Clin Dev Immunol 2012, 12. 10.1155/2012/720768 PMC3514849 23227088

[mol212095-bib-0037] Halama N , Zoernig I , Berthel A , Kahlert C , Klupp F , Suarez‐Carmona M , Suetterlin T , Brand K , Krauss J , Lasitschka F *et al* (2016) Tumoral immune cell exploitation in colorectal cancer metastases can be targeted effectively by anti‐CCR5 therapy in cancer patients. Cancer Cell 29, 587–601. 27070705 10.1016/j.ccell.2016.03.005

[mol212095-bib-0038] Hu Y , He MY , Zhu LF , Yang CC , Zhou ML , Wang Q , Zhang W , Zheng YY , Wang DM , Xu ZQ *et al* (2016) Tumor‐associated macrophages correlate with the clinicopathological features and poor outcomes via inducing epithelial to mesenchymal transition in oral squamous cell carcinoma. J Exp Clin Cancer Res 35, 12. 26769084 10.1186/s13046-015-0281-z PMC4714460

[mol212095-bib-0039] Hu P , Shen M , Zhang P , Zheng C , Pang Z , Zhu L and Du J (2015) Intratumoral neutrophil granulocytes contribute to epithelial‐mesenchymal transition in lung adenocarcinoma cells. Tumour Biol 36, 7789–7796. 25944163 10.1007/s13277-015-3484-1

[mol212095-bib-0040] Hwang WL , Yang MH , Tsai ML , Lan HY , Su SH , Chang SC , Teng HW , Yang SH , Lan YT , Chiou SH *et al* (2011) SNAIL regulates interleukin‐8 expression, stem cell‐like activity, and tumorigenicity of human colorectal carcinoma cells. Gastroenterology 141, 279–291. 21640118 10.1053/j.gastro.2011.04.008

[mol212095-bib-0041] Islam SA , Ling MF , Leung J , Shreffler WG and Luster AD (2013) Identification of human CCR8 as a CCL18 receptor. J Exp Med 210, 1889–1898. 23999500 10.1084/jem.20130240 PMC3782048

[mol212095-bib-0042] Izumi K , Fang LY , Mizokami A , Namiki M , Li L , Lin WJ and Chang C (2013) Targeting the androgen receptor with siRNA promotes prostate cancer metastasis through enhanced macrophage recruitment via CCL2/CCR2‐induced STAT3 activation. EMBO Mol Med 5, 1383–1401. 23982944 10.1002/emmm.201202367 PMC3799493

[mol212095-bib-0043] Jang MH , Kim HJ , Kim EJ , Chung YR and Park SY (2015) Expression of epithelial‐mesenchymal transition‐related markers in triple‐negative breast cancer: ZEB1 as a potential biomarker for poor clinical outcome. Hum Pathol 46, 1267–1274. 26170011 10.1016/j.humpath.2015.05.010

[mol212095-bib-0044] Ji S , Zhang W , Zhang X , Hao C , Hao A , Gao Q , Zhang H , Sun J and Hao J (2016) Sohlh2 suppresses epithelial to mesenchymal transition in breast cancer via downregulation of IL‐8. Oncotarget 7, 49411–49424. 27384482 10.18632/oncotarget.10355 PMC5226517

[mol212095-bib-0045] Jiang Y‐X , Yang S‐W , Li P‐A , Luo X , Li Z‐Y , Ho Y‐X and Yu P‐W (2017) The promotion of the transformation of quiescent gastric cancer stem cells by IL‐17 and the underlying mechanisms. Oncogene 36, 1256–1264. 27524415 10.1038/onc.2016.291 PMC5340802

[mol212095-bib-0046] Jiao L , Li D‐D , Yang C‐L , Peng R‐Q , Guo Y‐Q , Zhang X‐S and Zhu X‐F (2016) Reactive oxygen species mediate oxaliplatin‐induced epithelial‐mesenchymal transition and invasive potential in colon cancer. Tumor Biology 37, 8413–8423. 26733168 10.1007/s13277-015-4736-9

[mol212095-bib-0047] Johansson J , Tabor V , Wikell A , Jalkanen S and Fuxe J (2015) TGF‐beta1‐induced epithelial‐mesenchymal transition promotes monocyte/macrophage properties in breast cancer cells. Front Oncol 5, 3. 25674539 10.3389/fonc.2015.00003 PMC4306317

[mol212095-bib-0048] Kapur N , Mir H , Clark CE , Krishnamurti U , Beech DJ , Lillard JW and Singh S (2016) CCR6 expression in colon cancer is associated with advanced disease and supports epithelial‐to‐mesenchymal transition. Br J Cancer 114, 1343–1451. 27149649 10.1038/bjc.2016.113 PMC4984452

[mol212095-bib-0049] Kim MS , Jeong J , Seo J , Kim HS , Kim SJ and Jin W (2016) Dysregulated JAK2 expression by TrkC promotes metastasis potential, and EMT program of metastatic breast cancer. Sci Rep 6, 33899. 27654855 10.1038/srep33899 PMC5032000

[mol212095-bib-0050] Kudo‐Saito C , Shirako H , Ohike M , Tsukamoto N and Kawakami Y (2013) CCL2 is critical for immunosuppression to promote cancer metastasis. Clin Exp Metastasis 30, 393–405. 23143679 10.1007/s10585-012-9545-6

[mol212095-bib-0051] Kudo‐Saito C , Shirako H , Takeuchi T and Kawakami Y (2009) Cancer metastasis is accelerated through immunosuppression during Snail‐induced EMT of cancer cells. Cancer Cell 15, 195–206. 19249678 10.1016/j.ccr.2009.01.023

[mol212095-bib-0052] Lee CH , Chang JS , Syu SH , Wong TS , Chan JY , Tang YC , Yang ZP , Yang WC , Chen CT , Lu SC *et al* (2015) IL‐1beta promotes malignant transformation and tumor aggressiveness in oral cancer. J Cell Physiol 230, 875–884. 25204733 10.1002/jcp.24816

[mol212095-bib-0053] Lee SO , Yang X , Duan S , Tsai Y , Strojny LR , Keng P and Chen Y (2016) IL‐6 promotes growth and epithelial‐mesenchymal transition of CD133+ cells of non‐small cell lung cancer. Oncotarget 7, 6626–6638. 26675547 10.18632/oncotarget.6570 PMC4872738

[mol212095-bib-0054] Lesage J , Suarez‐Carmona M , Neyrinck‐Leglantier D , Grelet S , Blacher S , Hunziker W , Birembaut P , Noel A , Nawrocki‐Raby B , Gilles C *et al* (2017) Zonula occludens‐1/NF‐kappaB/CXCL8: a new regulatory axis for tumor angiogenesis. FASEB J 31, 1678–1688. 28057697 10.1096/fj.201600890R

[mol212095-bib-0055] Li S , Kendall SE , Raices R , Finlay J , Covarrubias M , Liu Z , Lowe G , Lin YH , Teh YH , Leigh V *et al* (2012a) TWIST1 associates with NF‐kappaB subunit RELA via carboxyl‐terminal WR domain to promote cell autonomous invasion through IL8 production. BMC Biol 10, 73. 22891766 10.1186/1741-7007-10-73 PMC3482588

[mol212095-bib-0056] Li Y , Wang L , Pappan L , Galliher‐Beckley A and Shi J (2012b) IL‐1β promotes stemness and invasiveness of colon cancer cells through Zeb1 activation. Mol Cancer 11, 13. 23174018 10.1186/1476-4598-11-87 PMC3532073

[mol212095-bib-0057] Lim S , Becker A , Zimmer A , Lu J , Buettner R and Kirfel J (2013) SNAI1‐mediated epithelial‐mesenchymal transition confers chemoresistance and cellular plasticity by regulating genes involved in cell death and stem cell maintenance. PLoS One 8, e66558. 23799116 10.1371/journal.pone.0066558 PMC3684605

[mol212095-bib-0058] Liu C‐Y , Xu J‐Y , Shi X‐Y , Huang W , Ruan T‐Y , Xie P and Ding JL (2013) M2‐polarized tumor‐associated macrophages promoted epithelial–mesenchymal transition in pancreatic cancer cells, partially through TLR4/IL‐10 signaling pathway. Lab Invest 93, 844–854. 23752129 10.1038/labinvest.2013.69

[mol212095-bib-0059] Long H , Xiang T , Qi W , Li Y , Huang J , Xiz R , Chen J and Zhu B (2015) CD133+ ovarian cancer stem‐like cells promote non‐stem cancer cell metastasis via CCL5 induced epithelial‐mesenchymal transition. Oncotarget 6, 5847–5860. 10.18632/oncotarget.3462 PMC4467406 25788271

[mol212095-bib-0060] Long X , Ye Y , Zhang L , Liu P , Yu W , Wei F , Ren X and Yu J (2016) IL‐8, a novel messenger to cross‐link inflammation and tumor EMT via autocrine and paracrine pathways (Review). Int J Oncol 48, 5–12. 26548401 10.3892/ijo.2015.3234

[mol212095-bib-0061] Lopez‐Novoa JM and Nieto MA (2009) Inflammation and EMT: an alliance towards organ fibrosis and cancer progression. EMBO Mol Med 1, 303–314. 20049734 10.1002/emmm.200900043 PMC3378143

[mol212095-bib-0062] Lv N , Gao Y , Guan H , Wu D , Ding S , Teng W and Shan Z (2015) Inflammatory mediators, tumor necrosis factor‐alpha and interferon‐gamma, induce EMT in human PTC cell lines. Oncol Lett 10, 2591–2597. 26622895 10.3892/ol.2015.3518 PMC4580000

[mol212095-bib-0063] Lyons JG , Patel V , Roue NC , Fok SY , Soon LL , Halliday GM and Gutkind JS (2008) Snail up‐regulates proinflammatory mediators and inhibits differentiation in oral keratinocytes. Cancer Res 68, 4525–4530. 18559496 10.1158/1078-0432.CCR-07-6735 PMC2631428

[mol212095-bib-0064] Malfettone A , Soukupova J , Bertran E , Crosas‐Molist E , Lastra R , Fernando J , Koudelkova P , Rani B , Fabra Á , Serrano T *et al* (2017) Transforming growth factor‐β‐induced plasticity causes a migratory stemness phenotype in hepatocellular carcinoma. Cancer Lett 392, 39–50. 28161507 10.1016/j.canlet.2017.01.037

[mol212095-bib-0065] Marcucci F , Stassi G and De Maria R (2016) Epithelial–mesenchymal transition: a new target in anticancer drug discovery. Nat Rev Drug Discovery 15, 311–325. 26822829 10.1038/nrd.2015.13

[mol212095-bib-0066] Meng F , Li W , Li C , Gao Z , Guo K and Song S (2015) CCL18 promotes epithelial‐mesenchymal transition, invasion and migration of pancreatic cancer cells in pancreatic ductal adenocarcinoma. Int J Oncol 46, 1109–1120. 25502147 10.3892/ijo.2014.2794

[mol212095-bib-0067] Miao JW , Liu LJ and Huang J (2014) Interleukin‐6‐induced epithelial‐mesenchymal transition through signal transducer and activator of transcription 3 in human cervical carcinoma. Int J Oncol 45, 165–176. 24806843 10.3892/ijo.2014.2422

[mol212095-bib-0069] Moustakas A and Heldin C‐H (2016) Mechanisms of TGFβ‐induced epithelial‐mesenchymal transition. J Clin Med 5, 63. 10.3390/jcm5070063 PMC4961994 27367735

[mol212095-bib-0070] Mucha J , Majchrzak K , Taciak B , Hellmen E and Krol M (2014) MDSCs mediate angiogenesis and predispose canine mammary tumor cells for metastasis via IL‐28/IL‐28RA (IFN‐lambda) signaling. PLoS One 9, e103249. 25075523 10.1371/journal.pone.0103249 PMC4116234

[mol212095-bib-0071] Muller AJ , Sharma MD , Chandler PR , Duhadaway JB , Everhart ME , Johnson BA 3rd , Kahler DJ , Pihkala J , Soler AP , Munn DH *et al* (2008) Chronic inflammation that facilitates tumor progression creates local immune suppression by inducing indoleamine 2,3 dioxygenase. Proc Natl Acad Sci U S A 105, 17073–17078. 18952840 10.1073/pnas.0806173105 PMC2579380

[mol212095-bib-0072] Naugler WE and Karin M (2008) The wolf in sheep's clothing: the role of interleukin‐6 in immunity, inflammation and cancer. Trends Mol Med 14, 109–119. 18261959 10.1016/j.molmed.2007.12.007

[mol212095-bib-0073] Noman MZ , Janji B , Abdou A , Hasmim M , Terry S , Tan TZ , Mami‐Chouaib F , Thiery JP and Chouaib S (2017) The immune checkpoint ligand PD‐L1 is upregulated in EMT‐activated human breast cancer cells by a mechanism involving ZEB‐1 and miR‐200. Oncoimmunology 6, e1263412. 28197390 10.1080/2162402X.2016.1263412 PMC5283623

[mol212095-bib-0074] Pan Y , Guo X , Yang Z , Chen S , Lei Y , Lin M , Wang L , Feng C and Ke Z (2016) AEG‐1 activates Wnt/PCP signaling to promote metastasis in tongue squamous cell carcinoma. Oncotarget 7, 2093–2104. 26689985 10.18632/oncotarget.6573 PMC4811518

[mol212095-bib-0075] Pang MF , Georgoudaki AM , Lambut L , Johansson J , Tabor V , Hagikura K , Jin Y , Jansson M , Alexander JS , Nelson CM *et al* (2016) TGF‐[beta]1‐induced EMT promotes targeted migration of breast cancer cells through the lymphatic system by the activation of CCR7/CCL21‐mediated chemotaxis. Oncogene 35, 748–760. 25961925 10.1038/onc.2015.133 PMC4753256

[mol212095-bib-0076] Papageorgis P and Stylianopoulos T (2015) Role of TGFβ in regulation of the tumor microenvironment and drug delivery (Review). Int J Oncol 46, 933–943 25573346 10.3892/ijo.2015.2816 PMC4306018

[mol212095-bib-0077] Pesic M and Greten FR (2016) Inflammation and cancer: tissue regeneration gone awry. Curr Opin Cell Biol 43, 55–61. 27521599 10.1016/j.ceb.2016.07.010

[mol212095-bib-0078] Pickup M , Novitskiy S and Moses HL (2013) The roles of TGFbeta in the tumour microenvironment. Nat Rev Cancer 13, 788–799. 24132110 10.1038/nrc3603 PMC4025940

[mol212095-bib-0079] Pickup MW , Owens P and Moses HL (2017) TGF‐beta, bone morphogenetic protein, and activin signaling and the tumor microenvironment. Cold Spring Harb Perspect Biol, a022285. 10.1101/cshperspect.a022285 PMC5411701 28062564

[mol212095-bib-0080] Rao Q , Chen Y , Yeh C‐R , Ding J , Li L , Chang C and Yeh S (2016) Recruited mast cells in the tumor microenvironment enhance bladder cancer metastasis via modulation of ERβ/CCL2/CCR2 EMT/MMP9 signals. Oncotarget 7, 7842–7855. 26556868 10.18632/oncotarget.5467 PMC4884958

[mol212095-bib-0081] Ravi J , Elbaz M , Wani NA , Nasser MW and Ganju RK (2016) Cannabinoid receptor‐2 agonist inhibits macrophage induced EMT in non‐small cell lung cancer by downregulation of EGFR pathway. Mol Carcinog 55, 2063–2076. 26741322 10.1002/mc.22451 PMC7063844

[mol212095-bib-0082] de Reynies A , Jaurand MC , Renier A , Couchy G , Hysi I , Elarouci N , Galateau‐Salle F , Copin MC , Hofman P , Cazes A *et al* (2014) Molecular classification of malignant pleural mesothelioma: identification of a poor prognosis subgroup linked to the epithelial‐to‐mesenchymal transition. Clin Cancer Res 20, 1323–1334. 24443521 10.1158/1078-0432.CCR-13-2429

[mol212095-bib-0083] Ricciardi M , Zanotto M , Malpeli G , Bassi G , Perbellini O , Chilosi M , Bifari F and Krampera M (2015) Epithelial‐to‐mesenchymal transition (EMT) induced by inflammatory priming elicits mesenchymal stromal cell‐like immune‐modulatory properties in cancer cells. Br J Cancer 112, 1067–1075. 25668006 10.1038/bjc.2015.29 PMC4366889

[mol212095-bib-0084] Roepman P , Schlicker A , Tabernero J , Majewski I , Tian S , Moreno V , Snel MH , Chresta CM , Rosenberg R , Nitsche U *et al* (2014) Colorectal cancer intrinsic subtypes predict chemotherapy benefit, deficient mismatch repair and epithelial‐to‐mesenchymal transition. Int J Cancer 134, 552–562. 23852808 10.1002/ijc.28387 PMC4234005

[mol212095-bib-0085] Rokavec M , Oner MG , Li H , Jackstadt R , Jiang L , Lodygin D , Kaller M , Horst D , Ziegler PK , Schwitalla S *et al* (2014) IL‐6R/STAT3/miR‐34a feedback loop promotes EMT‐mediated colorectal cancer invasion and metastasis. J Clin Investig 124, 1853–1867. 24642471 10.1172/JCI73531 PMC3973098

[mol212095-bib-0086] Sangaletti S , Tripodo C , Santangelo A , Castioni N , Portararo P , Gulino A , Botti L , Parenza M , Cappetti B , Orlandi R *et al* (2016) Mesenchymal transition of high‐grade breast carcinomas depends on extracellular matrix control of myeloid suppressor cell activity. Cell Rep 17, 233–248. 27681434 10.1016/j.celrep.2016.08.075

[mol212095-bib-0087] Shagieva G , Domnina L , Makarevich O , Chernyak B , Skulachev V and Dugina V (2016) Depletion of mitochondrial reactive oxygen species downregulates epithelial‐to‐mesenchymal transition in cervical cancer cells. Oncotarget 8, 4901–4913. 10.18632/oncotarget.13612 PMC5354879 27902484

[mol212095-bib-0088] Shintani Y , Fujiwara A , Kimura T , Kawamura T , Funaki S , Minami M and Okumura M (2016) IL‐6 secreted from cancer‐associated fibroblasts mediates chemoresistance in NSCLC by increasing epithelial‐mesenchymal transition signaling. J Thorac Oncol 11, 1482–1492. 27287412 10.1016/j.jtho.2016.05.025

[mol212095-bib-0089] Solinas G , Germano G , Mantovani A and Allavena P (2009) Tumor‐associated macrophages (TAM) as major players of the cancer‐related inflammation. J Leukoc Biol 86, 1065–1073. 19741157 10.1189/jlb.0609385

[mol212095-bib-0090] Song J , Feng L , Zhong R , Xia Z , Zhang L , Cui L , Yan H , Jia X and Zhang Z (2016) Icariside II inhibits the EMT of NSCLC cells in inflammatory microenvironment via down‐regulation of Akt/NF‐kappaB signaling pathway. Mol Carcinog 56, 36–48. 26859114 10.1002/mc.22471

[mol212095-bib-0091] Spaderna S , Schmalhofer O , Hlubek F , Berx G , Eger A , Merkel S , Jung A , Kirchner T and Brabletz T (2006) A transient, EMT‐linked loss of basement membranes indicates metastasis and poor survival in colorectal cancer. Gastroenterology 131, 830–840. 16952552 10.1053/j.gastro.2006.06.016

[mol212095-bib-0092] Su S , Liu Q , Chen J , Chen J , Chen F , He C , Huang D , Wu W , Lin L , Huang W *et al* (2014) A positive feedback loop between mesenchymal‐like cancer cells and macrophages is essential to breast cancer metastasis. Cancer Cell 25, 605–620. 24823638 10.1016/j.ccr.2014.03.021

[mol212095-bib-0093] Suarez‐Carmona M , Bourcy M , Lesage J , Leroi N , Syne L , Blacher S , Hubert P , Erpicum C , Foidart JM , Delvenne P *et al* (2015) Soluble factors regulated by epithelial‐mesenchymal transition mediate tumour angiogenesis and myeloid cell recruitment. J Pathol 236, 491–504. 25880038 10.1002/path.4546

[mol212095-bib-0094] Subramani R , Gonzalez E , Arumugam A , Nandy S , Gonzalez V , Medel J , Camacho F , Ortega A , Bonkoungou S , Narayan M *et al* (2016) Nimbolide inhibits pancreatic cancer growth and metastasis through ROS‐mediated apoptosis and inhibition of epithelial‐to‐mesenchymal transition. Sci Rep 6, 19819. 26804739 10.1038/srep19819 PMC4726267

[mol212095-bib-0095] Sullivan NJ , Sasser AK , Axel AE , Vesuna F , Raman V , Ramirez N , Oberyszyn TM and Hall BM (2009) Interleukin‐6 induces an epithelial‐mesenchymal transition phenotype in human breast cancer cells. Oncogene 28, 2940–2947. 19581928 10.1038/onc.2009.180 PMC5576031

[mol212095-bib-0096] Sun KH , Sun GH , Wu YC , Ko BJ , Hsu HT and Wu ST (2016) TNF‐alpha augments CXCR2 and CXCR3 to promote progression of renal cell carcinoma. J Cell Mol Med 20, 2020–2028. 27297979 10.1111/jcmm.12890 PMC5082409

[mol212095-bib-0097] Toh B , Wang X , Keeble J , Sim WJ , Khoo K , Wong WC , Kato M , Prevost‐Blondel A , Thiery JP and Abastado JP (2011) Mesenchymal transition and dissemination of cancer cells is driven by myeloid‐derived suppressor cells infiltrating the primary tumor. PLoS Biol 9, e1001162. 21980263 10.1371/journal.pbio.1001162 PMC3181226

[mol212095-bib-0098] Ugel S , De Sanctis F , Mandruzzato S and Bronte V (2015) Tumor‐induced myeloid deviation: when myeloid‐derived suppressor cells meet tumor‐associated macrophages. J Clin Investig 125, 3365–3376. 26325033 10.1172/JCI80006 PMC4588310

[mol212095-bib-0099] Uyttenhove C , Pilotte L , Theate I , Stroobant V , Colau D , Parmentier N , Boon T and Van den Eynde BJ (2003) Evidence for a tumoral immune resistance mechanism based on tryptophan degradation by indoleamine 2,3‐dioxygenase. Nat Med 9, 1269–1274. 14502282 10.1038/nm934

[mol212095-bib-0100] Visciano C , Liotti F , Prevete N , Cali G , Franco R , Collina F , de Paulis A , Marone G , Santoro M and Melillo RM (2015) Mast cells induce epithelial‐to‐mesenchymal transition and stem cell features in human thyroid cancer cells through an IL‐8‐Akt‐Slug pathway. Oncogene 34, 5175–5186. 25619830 10.1038/onc.2014.441

[mol212095-bib-0101] Wang Q , Tang Y , Yu H , Yin Q , Li M , Shi L , Zhang W , Li D and Li L (2016) CCL18 from tumor‐cells promotes epithelial ovarian cancer metastasis via mTOR signaling pathway. Mol Carcinog 55, 1688–1699. 26457987 10.1002/mc.22419 PMC5057350

[mol212095-bib-0102] Wu D , Cheng J , Sun G , Wu S , Li M , Gao Z , Zhai S , Li P , Su D and Wang X (2016a) p70S6K promotes IL‐6‐induced epithelial‐mesenchymal transition and metastasis of head and neck squamous cell carcinoma. Oncotarget 7, 36539–36550. 27174914 10.18632/oncotarget.9282 PMC5095019

[mol212095-bib-0103] Wu YC , Tang SJ , Sun GH and Sun KH (2016b) CXCR7 mediates TGFbeta1‐promoted EMT and tumor‐initiating features in lung cancer. Oncogene 35, 2123–2132. 26212008 10.1038/onc.2015.274

[mol212095-bib-0104] Yadav A , Kumar B , Datta J , Teknos TN and Kumar P (2011) IL‐6 promotes head and neck tumor metastasis by inducing epithelial‐mesenchymal transition via the JAK‐STAT3‐SNAIL signaling pathway. Mol Cancer Res 9, 1658–1667. 21976712 10.1158/1541-7786.MCR-11-0271 PMC3243808

[mol212095-bib-0105] Yan Y , Zhang J , Li J‐H , Liu X , Wang J‐Z , Qu H‐Y , Wang J‐S and Duan X‐Y (2016) High tumor‐associated macrophages infiltration is associated with poor prognosis and may contribute to the phenomenon of epithelial–mesenchymal transition in gastric cancer. Onco Targets Ther 9, 3975–3983. 27418840 10.2147/OTT.S103112 PMC4935103

[mol212095-bib-0106] Ye LY , Chen W , Bai XL , Xu XY , Zhang Q , Xia XF , Sun X , Li GG , Hu QD , Fu QH *et al* (2016) Hypoxia‐induced epithelial‐to‐mesenchymal transition in hepatocellular carcinoma induces an immunosuppressive tumor microenvironment to promote metastasis. Cancer Res 76, 818–830. 26837767 10.1158/0008-5472.CAN-15-0977

[mol212095-bib-0107] Yin J , Zeng F , Wu N , Kang K , Yang Z and Yang H (2015) Interleukin‐8 promotes human ovarian cancer cell migration by epithelial‐mesenchymal transition induction in vitro. Clin Transl Oncol 17, 365–370. 25373532 10.1007/s12094-014-1240-4

[mol212095-bib-0108] Yu M , Bardia A , Wittner BS , Stott SL , Smas ME , Ting DT , Isakoff SJ , Ciciliano JC , Wells MN , Shah AM *et al* (2013) Circulating breast tumor cells exhibit dynamic changes in epithelial and mesenchymal composition. Science 339, 580–584. 23372014 10.1126/science.1228522 PMC3760262

[mol212095-bib-0109] Yu S , Yan C , Yang X , He S , Liu J , Qin C , Huang C , Lu Y , Tian Z and Jia L (2016) Pharmacoproteomic analysis reveals that metapristone (RU486 metabolite) intervenes E‐cadherin and vimentin to realize cancer metastasis chemoprevention. Sci Rep 6, 22388. 26932781 10.1038/srep22388 PMC4773818

[mol212095-bib-0110] Zhang Q , Liu S , Parajuli KR , Zhang W , Zhang K , Mo Z , Liu J , Chen Z , Yang S , Wang AR *et al* (2016d) Interleukin‐17 promotes prostate cancer via MMP7‐induced epithelial‐to‐mesenchymal transition. Oncogene 36, 687–699. 27375020 10.1038/onc.2016.240 PMC5213194

[mol212095-bib-0111] Zhang RL , Peng LX , Yang JP , Zheng LS , Xie P , Wang MY , Huang BJ , Zhao HR , Bao YX and Qian CN (2016e) IL‐8 suppresses E‐cadherin expression in nasopharyngeal carcinoma cells by enhancing E‐cadherin promoter DNA methylation. Int J Oncol 48, 207–214. 26530812 10.3892/ijo.2015.3226

[mol212095-bib-0112] Zhang J , Tian X‐J and Xing J (2016a) Signal transduction pathways of EMT induced by TGF‐β, SHH, and WNT and their crosstalks. J Clin Med 5, 41. 10.3390/jcm5040041 PMC4850464 27043642

[mol212095-bib-0113] Zhang L , Wang D , Li Y , Liu Y , Xie X , Wu Y , Zhou Y , Ren J , Zhang J , Zhu H *et al* (2016c) CCL21/CCR7 axis contributed to CD133(+) pancreatic cancer stem‐like cell metastasis via EMT and Erk/NF‐κB pathway. PLoS One 11, e0158529. 27505247 10.1371/journal.pone.0158529 PMC4978474

[mol212095-bib-0114] Zhang J , Yan Y , Yang Y , Wang L , Li M , Wang J , Liu X , Duan X and Wang J (2016b) High infiltration of tumor‐associated macrophages influences poor prognosis in human gastric cancer patients, associates with the phenomenon of EMT. Medicine 95, 1–6. 10.1097/MD.0000000000002636 PMC4753880 26871785

[mol212095-bib-0115] Zhao Z , Cheng X , Wang Y , Han R , Li L , Xiang T , He L , Long H , Zhu B and He Y (2014) Metformin inhibits the IL‐6‐induced epithelial‐mesenchymal transition and lung adenocarcinoma growth and metastasis. PLoS One 9, e95884. 24789104 10.1371/journal.pone.0095884 PMC4005743

[mol212095-bib-0116] Zheng X , Carstens JL , Kim J , Scheible M , Kaye J , Sugimoto H , Wu CC , LeBleu VS and Kalluri R (2015) Epithelial‐to‐mesenchymal transition is dispensable for metastasis but induces chemoresistance in pancreatic cancer. Nature 527, 525–530. 26560028 10.1038/nature16064 PMC4849281

[mol212095-bib-0117] Zhu Y , Cheng Y , Guo Y , Chen J , Chen F , Luo R and Li A (2016) Protein kinase D2 contributes to TNF‐alpha‐induced epithelial mesenchymal transition and invasion via the PI3K/GSK‐3beta/beta‐catenin pathway in hepatocellular carcinoma. Oncotarget 7, 5327–5341. 26683365 10.18632/oncotarget.6633 PMC4868689

